# The dataset of the lichen collection "Abramo Massalongo" preserved at the Natural History Museum of Venice

**DOI:** 10.3897/BDJ.14.e184587

**Published:** 2026-03-26

**Authors:** Stefano Martellos, Linda Seggi, Raffaella Trabucco

**Affiliations:** 1 University of Trieste, Department of Life Sciences, Trieste, Italy University of Trieste, Department of Life Sciences Trieste Italy; 2 Centro Interuniversitario per le Biodiversità Vegetale Big Data—PLANT DATA, Department of Biological, Geo-logical and Environmental Sciences, Alma Mater Studiorum University, Bologna, Italy Centro Interuniversitario per le Biodiversità Vegetale Big Data—PLANT DATA, Department of Biological, Geo-logical and Environmental Sciences, Alma Mater Studiorum University Bologna Italy; 3 Fondazione Musei Civici di Venezia, Natural History Museum of Venice Giancarlo Ligabue, Venice, Italy Fondazione Musei Civici di Venezia, Natural History Museum of Venice Giancarlo Ligabue Venice Italy

**Keywords:** digitisation, exsiccata, historical collections, mobilisation, specimens

## Abstract

**Background:**

Italy was one of the most active countries in the field of lichenology in the nineteenth century. Researchers such as Baglietto, De Notaris, Massalongo and Trevisan produced relevant herbaria, which are (at least in part) still preserved today. The digitisation of at least some of the collections is allowing the mobilisation of information from these specimens and aggregating the metadata in national and global repositories.

**New information:**

This dataset contains the metadata and images of 594 specimens for 490 infraspecific taxa belonging to 31 orders, 73 families and 218 genera, which were mostly collected in north-eastern Italy between 1845 and 1856. The digitisation was carried out following an image to data to web workflow. Specimens where digitally imaged, taking a panoramic view and one or more details. Data were standardised to Darwin Core, with image metadata standardised according to the Simple Multimedia extension.

## Introduction

### Abramo Massalongo

Abramo Bartolomeo Massalongo (13 May 1824 - 25 May 1860, Tregnago, Verona, NE Italy), had a wide array of scientific interests, ranging from palaeontology to botany. As a botanist, he became one of the most relevant lichenologists in Europe (Lazzarin 1991). He created a new system of classification of lichens, based mostly on the microscopic features of the spores (Nimis 2016). He was an extremely prolific researcher, with 30 publications on lichenology between 1850 and 1860. *Ricerche sulla autonomia dei licheni crostosi*, published in 1852, is one of his most famous works and, to the present day, continues to be a reference for researchers. Many taxa he described are still valid today and several taxa were named after him by lichenologists worldwide (Nimis 2016). Along with numerous publications, several natural history collections remain as a memento of his intense scientific activity. His collection of *exsiccata*, the *Lichenes Italici Exsiccati*, is preserved in a more or less complete form in various museums and universities around the world. Most of his findings, notes, publications and materials are preserved at the Museum of Natural History of Verona (Martinati and De Betta 1860, Di Carlo and Burato 2019). The Museum of Natural History of Venice "Giancarlo Ligabue" preserves a complete copy of the *Lichenes Italici Exsiccati*, in its original form, as well as the so far unpublished collection presented in this paper.

### The Collection

**Figure 1.**
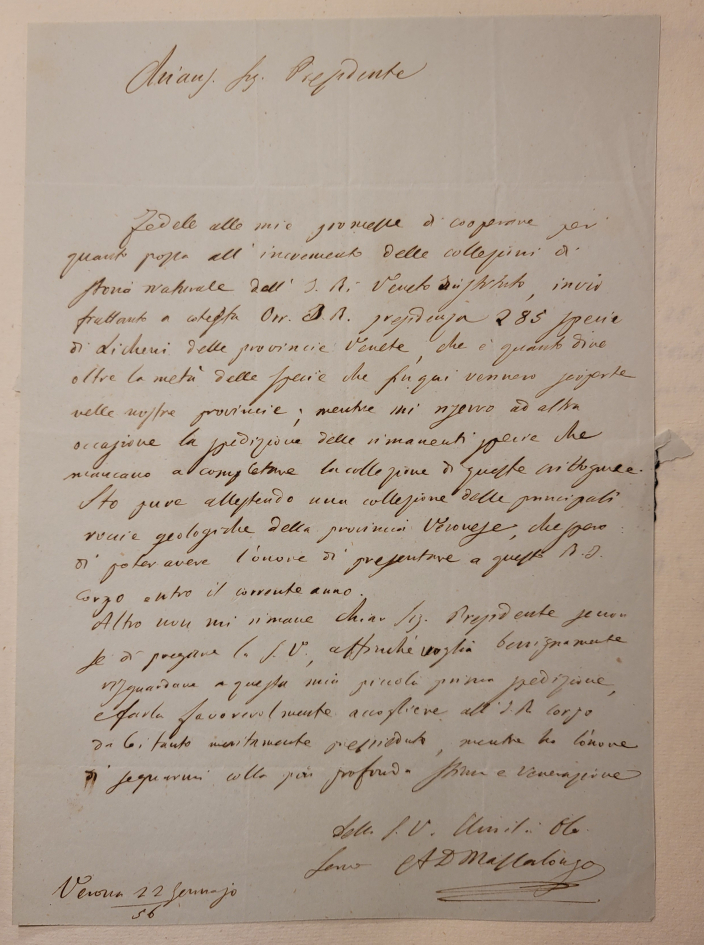
Letter from Abramo Massalongo to the President of the *Istituto Veneto di Scienze, Lettere ed Arti*, which accompanied the first batch of lichens donated by Massalongo himself (Istituto Veneto di Scienze, Lettere ed Arti; License CC BY).

**Figure 2.**
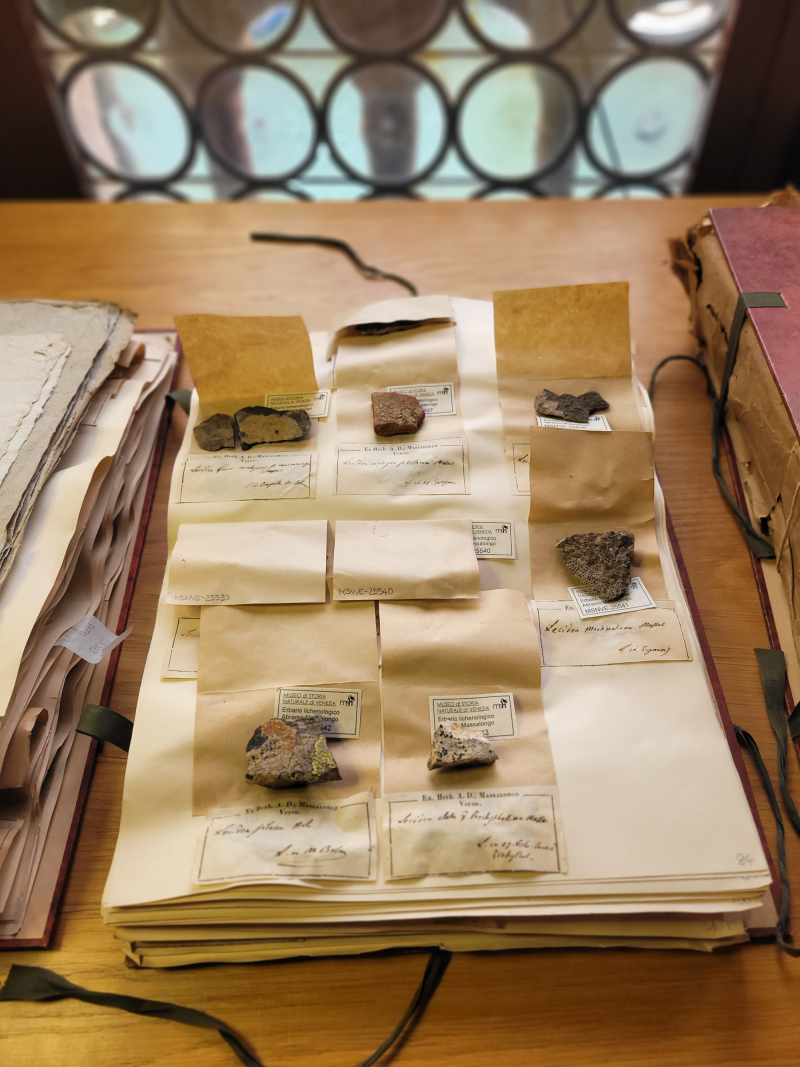
A sheet with some exsiccata of the collection.

The lichen collection "Abramo Massalongo" includes 594 *exsiccata* of 490 taxa, mainly collected in north-eastern Italy between 1845 and 1856. The specimens were collected for the most part by Massalongo himself and, to a minor extent, by other contributors. The collection was donated in at least two batches by Massalongo to the *Istituto Veneto di Scienze, Lettere ed Arti*, as specified in a letter dated 22 January 1856 (Fig. 1). From here, it was accessioned at the Museum of Natural History in 1923 along with other scientific collections. The collection consists of four issues containing 231 loose herbarium sheets and was probably arranged by Michelangelo Minio, director of the Museum from 1923 to 1947 (Fig. 2). The specimens are ordered alphabetically by genus and separated by substrate. Two issues contain lichens collected “on bark and soil”, while the other volumes contain lichens collected “on rock and bricks”. Each sheet hosts one to many specimens, either placed in envelopes or glued on to cardboard, sometimes folded for preservation purposes. All specimens are identified at least at the species level. The taxon name is handwritten near the specimens, together with information, such as the locality of collection, while the year of collection is present for a minority of specimens only. Information on synonyms, bibliographic references, substrate type, abundance and reproductive stage are sometimes reported as well. If the collector was not Massalongo, the name of the correspondent who sent the specimen, the herbarium of origin and the collector are reported as well.

### Digitisation

**Figure 3.**
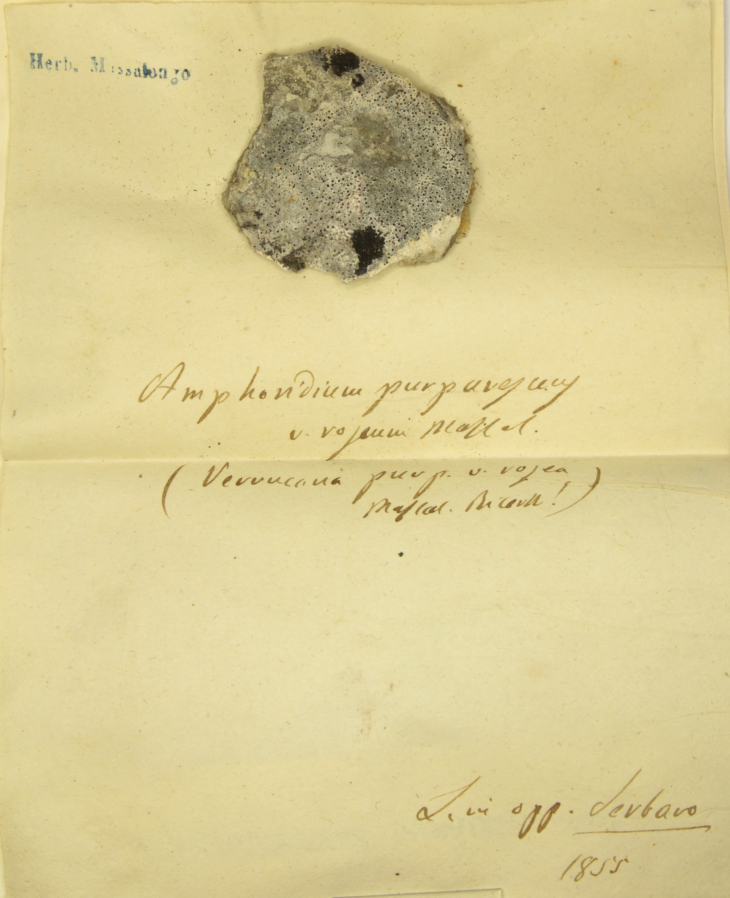
Specimen of Amphoridium purpurascens var. roseum A. Massal. with the original annotations of the author, as well as his stamp.

In recent years, the digitisation and mobilisation of natural history specimen data has become pivotal (Hedrick et al. 2020). The Natural History Museum of Venice "Giancarlo Ligabue" is currently carrying out digitisation of their collections, starting from those of historical relevance. This has led to the digitisation of the *Lichenotheca Veneta* by Vittore Trevisan (Martellos et al. 2024), another collection of lichens and the Algarium by Aristocle Vatova and Victor Schiffner (Seggi et al. 2024). These efforts are being coupled with the publication of the datasets (Martellos et al. 2025), after a careful revision of the data and of the nomenclature, in the Global Biodiverisity Information Facility (GBIF 2025). The digitisation of the lichen collection "Abramo Massalongo" was carried out following an image to data to web workflow. All the specimens where digitally imaged, taking at least a panoramic view of the specimens and the original writings of Massalongo, who normally wrote his annotations not on a label, but on the herbarium sheet itself or on the envelope of the specimen (Fig. 3). Furthermore, for all the specimens, one or more detail images of the fruiting bodies or of other structures which could be relevant for researchers, has been produced. Specimen data were standardised to Darwin Core (Wieczorek et al. 2012). Data and images have also been made available online (AA.VV. 2025) in a dedicated web portal.

Images and metadata, together with a description of the collection and some information about the authors have also been made available online at the URL: https://dryades.units.it/MUVE_Massalongo/index.html.

## Sampling methods

### Study extent

Almost all the specimens were collected by Abramo Massalongo, mostly in the area of the modern Veneto administrative region (NE Italy) and especially in the area north-east of Verona, close to his home village, Tregnago.

### Sampling description

The sampling strategy was preferential, since the author collected the specimens he considered relevant only. All the specimens were dried, glued to paper sheets or stored in paper envelopes and preserved.

## Geographic coverage

### Description

**Figure 4.**
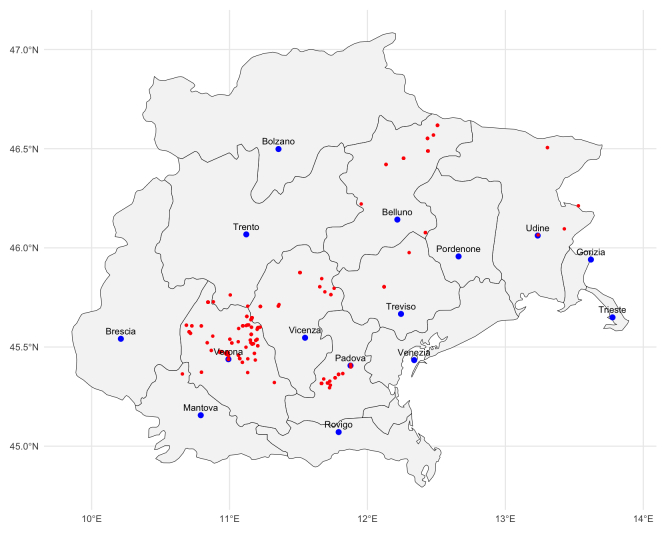
Map of the collection sites.

A map of the survey area is shown in Fig. 4. Amongst the specimens, those which have the locality of collection are 549 out of a total of 594. The 45 specimens lacking the locality of collection have no or poor information on the locality of collection, such as a generic "Italy"; thus, georeferencing them is a futile exercise. Those with an indication of the locality of collection were georeferenced *a posteriori*, using Google APIs (Google 2025) in the R environment by means of the function *geocode* in the package *ggmap* (Kahle and Wickham 2013), together with the maps produced by the IGMI - Istituto Geografico Militare Italiano (Italian Military Geographic Institute). All the localities where also double-checked manually, with the support of the original literature of Abramo Massalongo and of Arcanum Maps (Arcanum Maps 2025). The uncertainty of the point was estimated using the bounding box vertexes. As a practical approximation, the geocoder’s bounding box can be treated as an uncertainty zone, even if bounding boxes are not the same as formal uncertainty, since they represent either a feature’s polygon or a viewport suggested by the geocoder, not an actual measurement error. However, it is feasible to compute a radius starting from the centroid of the bounding box and the maximum great-circle distance from that centroid to the box corners. The radius (calculated in metres) is a conservative estimate of the actual uncertainty. The calculation was made in the R environment by means of the *distHaversine* command from the *geosphere* package (Hijmans 2025). The distHaversine command computes the shortest distance between two points, according to the 'haversine method’, which assumes a spherical earth, ignoring ellipsoidal effects (Sinnott 1984).

### Coordinates

45.29452 and 49.47053 Latitude; 10.65798 and 13.52939 Longitude.

## Taxonomic coverage

### Description

The collection contains 594 specimens for 490 infraspecific taxa, belonging to 31 orders, 73 families and 218 genera (Table 1). The nomenclature, including orders and families, was mostly aligned with the taxonomic backbone of ITALIC, the information system on Italian lichens (Martellos et al. 2023, Nimis 2025). When not possible, the authors used as a reference the portal *Index Fungorum* (Agerer et al. 2000, Index Fungorum Partnership 2025). In a very few specimens, however, it was impossible to align the names by Massalongo to modern nomenclature. As far as these specimens are concerned, only a critical revision of the specimens could lead to a complete understanding of the taxonomic delimitations by Massalongo. In these rare cases, the scientific name is the verbatim name, written according to the modern rules of taxonomic nomenclature. As an example, the verbatim name Amphoridium purpurescens v. roseum Massal. (Fig. 3) was corrected into Amphoridium purpurascens var. roseum A. Massal. Even if *Amphoridium purpurascens* A. Massal. is currently treated as a synonym of Bagliettoa marmorea (Scop.) Gueidan & Cl. Roux, its var. roseum is not present in any nomenclatory repository and, thus, the name was reported as originally conceived by Massalongo.

The complete list of genera (with the number of infrageneric taxa) is: *Abrothallus* (2); *Acarospora* (8); *Acolium* (1); *Acrocordia* (2); *Alectoria* (3); *Alyxoria* (3); *Amphoridium* (1); *Anaptychia* (4); *Anisomeridium* (1); *Arthonia* (5); *Arthopyrenia* (11); *Arthrorhaphis* (1); *Aspicilia* (2); *Bacidia* (3); *Baeomyces* (1); *Bagliettoa* (4); *Bellicidia* (2); *Biatora* (2); *Biatorella* (1); *Biatorina* (1); *Bilimbia* (3); *Blastenia* (6); *Blastodesmia* (1); *Blennothallia* (1); *Brodoa* (3); *Bryobilimbia* (1); *Bryoplaca* (2); *Bryoria* (1); *Buellia* (6); *Calicium* (6); *Callopisma* (2); *Calogaya* (1); *Caloplaca* (3); *Candelaria* (3); *Candelariella* (2); *Catillaria* (2); *Cerothallia* (2); *Cetraria* (7); *Circinaria* (5); *Cladonia* (21); *Cliostomum* (1); *Coenogonium* (1); *Collema* (6); *Collemopsis* (1); *Coniangium* (1); *Coniocarpon* (2); *Coniocybe* (1); *Cornicularia* (1); *Cyphelium* (1); *Cyrtidula* (2); *Dendrographa* (1); *Dermatocarpon* (4); *Dibaeis* (2); *Didymella* (1); *Diploschistes* (7); *Diplotomma* (5); *Dirina* (1); *Dolichousnea* (1); *Enchylium* (5); *Endocarpon* (3); *Eopyrenula* (1); *Ephebe* (1); *Evernia* (3); *Flavoparmelia* (1); *Flavoplaca* (1); *Forssellia* (2); *Fuscidea* (1); *Glaucomaria* (1); *Graphis* (4); *Gyalecta* (6); *Gyalolechia* (5); *Haematomma* (2); *Heppia* (1); *Heterodermia* (1); *Huneckia* (3); *Hymenelia* (3); *Hyperphyscia* (1); *Hypogymnia* (1); *Hypotrachyna* (1); *Icmadophila* (1); *Ingvariella* (1); *Inoderma* (1); *Julella* (1); *Koerberia* (1); *Kuettlingeria* (2); *Lathagrium* (4); *Lecania* (3); *Lecanographa* (1); *Lecanora* (18); *Lecanoropsis* (1); *Lecidea* (8); *Lecidella* (5); *Lecidoma* (1); *Lempholemma* (1); *Lepra* (6); *Lepraria* (1); *Leprocaulon* (1); *Leptogium* (4); *Leptorhaphis* (1); *Letharia* (1); *Lobaria* (3); *Lobarina* (2); *Lobothallia* (3); *Lopadium* (1); *Maronea* (1); *Massalongia* (1); *Megaspora* (1); *Melanelia* (3); *Melanelixia* (1); *Melanohalea* (4); *Melaspilea* (1); *Menegazzia* (1); *Micarea* (2); *Microlecia* (2); *Multiclavula* (1); *Mycoblastus* (2); *Myriangium* (1); *Naetrocymbe* (5); *Naevia* (4); *Nephroma* (3); *Nephromopsis* (2); *Nesothele* (1); *Normandina* (1); *Ochrolechia* (4); *Opegrapha* (4); *Ophioparma* (2); *Pannaria* (2); *Parabagliettoa* (4); *Parmelia* (2); *Parmeliella* (2); *Parmelina* (2); *Parmeliopsis* (1); *Parmotrema* (2); *Peltigera* (8); *Peltula* (1); *Pertusaria* (8); *Petractis* (2); *Phaeophyscia* (1); *Physcia* (6); *Physciella* (1); *Physconia* (1); *Placidium* (1); *Placocarpus* (1); *Placodium* (1); *Placolecis* (2); *Placopyrenium* (1); *Placynthium* (4); *Platismatia* (1); *Plectocarpon* (1); *Pleurosticta* (2); *Polyblastia* (1); *Polyozosia* (7); *Porpidia* (5); *Porpidinia* (2); *Pragmopora* (1); *Protoblastenia* (6); *Protopannaria* (1); *Protoparmeliopsis* (2); *Pseudevernia* (2); *Pseudoschismatomma* (2); *Psora* (3); *Psoroma* (3); *Psorotichia* (1); *Punctelia* (1); *Pycnothelia* (1); *Pyrenodesmia* (5); *Pyrenula* (4); *Pyrrhospora* (1); *Ramalina* (7); *Rebentischia* (1); *Rehmia* (3); *Rhizocarpon* (2); *Rhizoplaca* (1); *Ricasolia* (3); *Rinodina* (5); *Rinodinella* (2); *Romjularia* (2); *Rusavskia* (1); *Sagedia* (1); *Sanguineodiscus* (2); *Sarcogyne* (1); *Sclerophora* (1); *Scoliciosporum* (1); *Scytinium* (3); *Solorina* (3); *Sparria* (3); *Sphaerophorus* (2); *Sphinctrina* (1); *Sporastatia* (1); *Squamarina* (8); *Squamulea* (1); *Staurothele* (3); *Stereocaulon* (7); *Sticta* (2); *Swinscowia* (3); *Synarthonia* (2); *Teloschistes* (2); *Tephromela* (1); *Tetramelas* (1); *Thalloidima* (8); *Thecaria* (1); *Thelidium* (2); *Thelotrema* (1); *Thrombium* (2); *Tomasellia* (2); *Toninia* (6); *Trapelia* (1); *Trapeliopsis* (2); *Umbilicaria* (4); *Usnea* (4); *Vahliella* (2); *Variospora* (4); *Verrucaria* (21); *Verruculopsis* (1); *Xalocoa* (2); *Xanthocarpia* (2); *Xanthoparmelia* (4); *Xanthoria* (3).

## Temporal coverage

**Data range:** 1845-1-01 – 1856-1-22.

### Notes

All the specimens by Abramo Massalongo were collected between 1850 and 1856. Even if most of the specimens lack information on the date of collection, this can be easily inferred by the period of activity of Massalongo on lichens, which, according to De Visiani (De Visiani 1861), began in 1850 and from the date of donation of the collection to the *Istituto Veneto di Scienze, Lettere ed Arti* (22 January 1856, see Fig. 1). As far as the specimens collected by other contributors are concerned, it is impossible to infer the date of collection when not reported on the labels. However, it can be assumed they were collected in the same range of time or slightly before, given that most of the contributors were contempories of Massalongo. One specimen annotation only reports an earlier date of collection (1845). However, there is not the name of the collector on the label. Given that the interest for lichens of Massalongo is reported to have arisen around the year 1850 (De Visiani 1861), it is possible that this specimen was provided to him by an anonymous contributor or that it was collected by Massalongo himself at the very beginning of his activity as a naturalist, when, in 1844, he switched his interests from medicine to botany (De Visiani 1861).

## Usage licence

### Usage licence

Creative Commons Public Domain Waiver (CC-Zero)

## Data resources

### Data package title

Abramo Massalongo Lichen Collection

### Resource link

https://doi.org/10.15468/6gcus5

### Alternative identifiers

https://www.gbif.org/dataset/80d45f89-a4ee-4697-af86-eea85d495ec4

### Number of data sets

1

### Data set 1.

#### Data set name

Abramo Massalongo Lichen Collection

#### Data format

Darwin Core

#### Download URL

https://www.gbif.org/dataset/80d45f89-a4ee-4697-af86-eea85d495ec4

#### Description

The lichen collection "Abramo Massalongo" includes 594 exsiccata of 490 taxa, mainly collected in north-eastern Italy between 1845 and 1855. The collection represents a historically and taxonomically significant dataset documenting early lichenological research in Italy and Europe.

**Data set 1.** 

Column label	Column description
occurrenceID	An identifier for the occurrence.
verbatimLabel	The verbatim transcription of the label or of the writings associated with the specimen on the herbarium sheet or envelope.
verbatimIdentification	The original identification of the organism, as written on the label.
identificationRemarks	Annotations on the original identification.
occurrenceRemarks	Annotations on the gathering event.
scientificName	Scientific name of the organism.
kingdom	Higher taxon - kingdom.
phylum	Higher taxon - phylum.
order	Higher taxon - order.
family	Higher taxon - family.
genus	The genus of the organism.
taxonRank	Taxonomic rank of the scientific name.
continent	The continent of the gathering event.
country	The country of the gathering event.
higherGeography	List of countries when it was impossible to associate the record to one country alone.
stateProvince	The province of the gathering event.
county	The county of the gathering event.
municipality	The municipality of the gathering event.
verbatimLocality	The locality of the gathering event, as originally written on the label.
decimalLatitude	Latitude of the location in decimal degrees.
decimalLongitude	Longitude of the location in decimal degrees.
coordinateUncertaintyInMetres	An estimation of the horizontal distance (in metres) from the point described by decimalLatitude and decimalLongitude, distance which describes the smallest circle containing the whole verbatimLocality.
geodeticDatum	The geodetic datum upon which the coordinates in decimalLatitude and decimalLongitude are based.
eventDate	Date on which the occurrence was recorded.
basisOfRecord	The specific nature of the data record.
institutionCode	The acronym of the institution having custody of the specimen referred to in the record.
datasetName	The name identifying the dataset from which the record is derived.
occurrenceStatus	A statement about whether the record is present or absent.

## Figures and Tables

**Table 1.** Distribution of the specimens in orders, families, genera and infrageneric taxa.

Abrothallales	1	1	2
Acarosporales	1	2	7
Arthoniales	4	14	22
Baeomycetales	2	3	4
Caliciales	2	15	33
Candelariales	2	2	3
Cantharellales	1	1	1
Coniocybales	1	2	2
Eremithallales	1	1	1
Helotiales	1	1	1
Hymeneliales	1	1	3
Incertae sedis	2	4	4
Lecanorales	14	62	147
Lecideales	3	8	16
Leprocaulales	1	1	1
Lichinales	3	7	7
Monoblastiales	1	2	3
Mycocaliciales	1	1	1
Myriangiales	1	1	1
Ostropales	5	9	15
Peltigerales	9	21	42
Pertusariales	4	10	22
Pleosporales	3	5	10
Pyrenulales	1	2	4
Rhizocarpales	2	3	3
Strigulales	1	1	2
Teloschistales	1	18	31
Trypetheliales	1	2	7
Tubeufiales	1	1	1
Umbilicariales	3	4	6
Verrucariales	1	15	32
